# Relation between Temperature and Mortality in Thirteen Spanish Cities

**DOI:** 10.3390/ijerph7083196

**Published:** 2010-08-11

**Authors:** Carmen Iñiguez, Ferran Ballester, Juan Ferrandiz, Santiago Pérez-Hoyos, Marc Sáez, Antonio López

**Affiliations:** 1 Center for Public Health Research (CSISP), Avda Catalunya 21, 46020, Valencia, Spain; E-Mail: inyigez_car@gva.es (C.I.); 2 Spanish Consortium for Research on Epidemiology and Public Health (CIBERESP), Doctor Aiguader, 88 1ªPlanta, 8003 Barcelona, Spain; 3 Valencian School for Studies on Health (EVES), Juan de Garay 21, 46017, Valencia, Spain; 4 University of Valencia, Jaume Roig s/n, 46010, Valencia, Spain; E-Mail: Antonio.Lopez@uv.es (A.L.); 5 Research Institute, Hospital Vall d’Hebrón, Passeig Vall d’Hebron, 119-129 08035 Barcelona, Spain; E-Mail: santi.perezhoyos@gmail.com (S.P.-H.); 6 Departament d’Economia, University of Girona, Campus de Montilivi 17071 Girona, Spain; E-Mail: marc.saez@udg.edu (M.S.)

**Keywords:** temperature, mortality, Spain

## Abstract

In this study we examined the shape of the association between temperature and mortality in 13 Spanish cities representing a wide range of climatic and socio-demographic conditions. The temperature value linked with minimum mortality (MMT) and the slopes before and after the turning point (MMT) were calculated. Most cities showed a V-shaped temperature-mortality relationship. MMTs were generally higher in cities with warmer climates. Cold and heat effects also depended on climate: effects were greater in hotter cities but lesser in cities with higher variability. The effect of heat was greater than the effect of cold. The effect of cold and MMT was, in general, greater for cardio-respiratory mortality than for total mortality, while the effect of heat was, in general, greater among the elderly.

## 1. Introduction

For many years, the effect of temperature on mortality has been the subject of numerous studies, mostly examining the impact of extreme weather events [1,2]. These studies have shown the existence of an association between temperature and mortality, but some characteristics such as the shape of the association remain less clear. The relationship between temperature and mortality usually displays a V or U-shaped pattern [3,4] (*i.e.*, populations experience increases in mortality due to both high and low temperatures), but other shapes like W [5] or J [6] have also been described. The shape of the association may vary widely from city to city because it depends on many factors such as climate (range of temperatures and atmospheric conditions to which the population has adapted), socio-economical level and age. Two conclusions may be drawn from this: on the one hand, studies incorporating information from several locations would be useful [6,7] and on the other, all available factors that might confound the relationship should be considered.

In recent years there has been growing concern about the possible effects of climatic changes including their effect on health [8,9]. Long term weather forecasts show that, if the tendency towards global warming continues, extreme meteorological phenomena such as heat waves or floods are likely to occur with higher intensity and frequency. These phenomena could have a greater impact on human health than that caused by the generalised temperature increase itself [10,11]. It is, therefore, of major interest to conduct studies which help to understand the complex relationships between meteorological factors and also contribute to the evaluation of surveillance and prevention measures [12].

We investigated the shape of the relationship between temperature and mortality in 13 Spanish cities representing a wide range of climatic, socio-demographic and environmental conditions. Our aims were: To determine the temperature at which mortality is lowest, to evaluate the impact of temperature changes below and above such value, to detect if there is any specificity of the effect for groups of causes or ages, and to identify possible common patterns among cities.

## 2. Material and Methods

**Figure 1.**
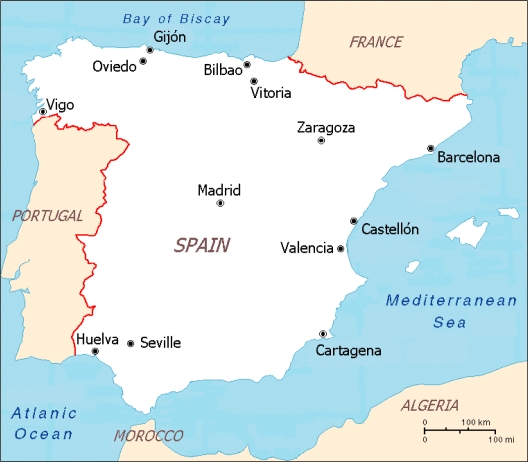
Spanish cities participating in the study.

In this study, we used data from the EMECAM project (Multicenter Study on Short-Term Effects of Air Pollution on Mortality) [13] including daily information on 13 Spanish cities: Barcelona, Bilbao, Cartagena, Castellón, Gijon, Huelva, Madrid, Oviedo, Seville, Valencia, Vigo, Vitoria and Zaragoza (Figure 1). The series covered variable study periods between 1990 and 1996, and always included at least three consecutive years. The data were obtained from public institution registers by the researcher responsible for each city, following a standardised protocol.

Mortality was computed as the daily number of deaths occurring in each city. All natural deaths (ICD-9: 0–799), deaths due to cardio-respiratory causes (ICD-9: 390–519), and deaths in people aged over 70 years were analysed. Daily mean temperature (average of the minimum and maximum values of current day) and daily mean humidity (average of the values at 0, 7, 13 and 18 hours in the current day) were obtained from the airport meteorological station located closest to the city centre and were provided by the Meteorological Service in each city (Table 1).

The following variables were considered as potential confounders: air pollution (level of suspended particulates measured as particulates of less than 10 μg in diameter (PM_10_) or, in its absence, black smoke (BS) or, in its absence, total suspended particulates (TSP)), daily incidence of influenza obtained for each day as 1/7 of the weekly value declared to the Compulsory Notifiable Disease Registry, and other calendar variables such as the day of the week, bank holiday and unusual events indicators.

The statistical model used to describe the relationship was the Poisson generalised additive model (GAM). This model was chosen as being the most suitable to explore the shape of a relationship. Its usefulness lies in the possibility of incorporating variables in a non-parametric way using smooth functions such as loess or spline, therefore avoiding the need to presuppose the shape of the relationship and later trying to reproduce it by means of an approximate functional expression. All analyses were carried out in S-Plus with stricter convergence criteria than default (tolerance = 10^−8^ and maximum number of iterations = 1,000) [14–16].

The models were constructed as follows: first, time since the beginning of the study smoothed by loess was introduced to control for secular trends and seasonality. The number between 90 and 360 (30 by 30) days that minimised the sum of the residual’s partial autocorrelation function (PACF) was chosen as span. After, a cubic smoothing spline of influenza cases previously smoothed by loess (with a 30 day span) was introduced. The number of degrees of freedom of this spline was 2 or 3 according to the minimum Akaike criterion [17]. Temperature and humidity were incorporated, each by means of three cubic smoothing splines with four degrees of freedom each. The first was the spline of current mean daily values, the second the spline of the linear regression residuals of the current values over lags from 1 to 3, and the third the spline of the linear regression residuals of the current values over lags from 4 to 10. This was done in an attempt to account for the lagged effect of both temperature and humidity, avoiding collinearity as far as possible [6]. Day of the week, air pollution (lags 0 and 1 average), holidays and special events, in this order, were linearly incorporated into the model if the likelihood ratio test was significant (p < 0.2). To account for serial correlation in the residuals where it remained in the final model, autoregressive terms were added into the model as appropriate.

Following this scheme, explicative models for each outcome and city were constructed. The significance of temperature was evaluated using the likelihood ratio test. The relationship was graphically represented and the current day temperature at which the curve achieved its minimum, the minimum mortality temperature (MMT), was obtained. Slopes below (cold slope) and above (heat slope) the MMT were estimated by linear regression of predicted mortality over temperature. The impact of cold and heat was expressed as the percentage change in mortality for a temperature change of 1 ºC.

## 3. Results and Discussion

### 3.1. Shape of the Relationship between Temperature and Mortality

The relationship between temperature and total mortality was significant in nine of the 13 cities, including the most populated. In the case of mortality due to specific causes and mortality in the elderly, the relationship was also significant in Madrid, Barcelona, Valencia, Seville and Zaragoza (five of the six most populated cities), and non-significant in Huelva and Cartagena (two of the three least populated cities).

**Figure 2.**
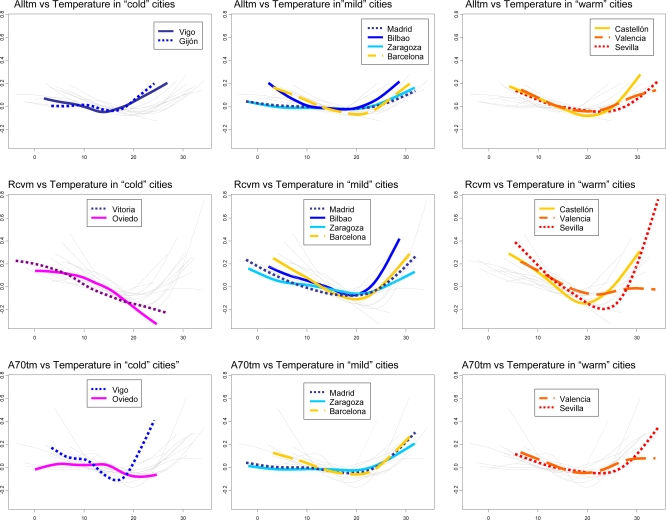
Curves of significant associations between temperature and mortality for: total mortality (Alltm) in the first row, cardio-respiratory mortality (Rcvm) in the second row, and mortality among people 70 years old or over (A70tm) in the third. Cities are grouped according to their mean temperature into “cold” cities (Vitoria, Oviedo, Vigo, Gijon), “mild” cities (Madrid, Bilbao, Zaragoza, Barcelona) and “warm” cities (Castellón, Valencia, Huelva, Seville, Cartagena). The rest of the curves are drawn in light gray.

Focusing on significant associations, the relationship between temperature and mortality was V or U-shaped (Figure 2), with largest effects (steeper slopes) for cardio-respiratory deaths. This shape does not suggest an acute effect of extreme temperatures but a rise in the death rate when the weather gets colder or hotter than the comfort band.

The only exception was the linear shape with a negative slope found in the two coldest cities (Vitoria and Oviedo). This shape could be explained by the narrow range of temperatures in these series and the lack of sufficient high temperatures to reveal the potential effect of heat on mortality due to circulatory causes: in Vitoria the temperature remained below 23 ºC for 95 % of the days of the series and in Oviedo below 20 ºC. Furthermore, both cities are small and reported a low number of deaths which could result in somewhat imprecise estimates. In fact, from a statistical point of view, one of the limitations of our study may be the low power when analysing series with a scarce number of events [18].

Grouping the cities with a significant relationship into three categories, “cold cities”, “mild cities” and “warm cities”, according to the daily mean temperature, allows for an appreciation of patterns between the curves. A relevant feature is the MMT deviation towards the right of each curve. Madrid and Zaragoza, classified as “mild cities”, showed a higher smoothening in both slopes. The other two “mild” cities: Bilbao and Barcelona are alike in their shape and different from the two previous cities.

This coincides with the similarities in climate and temperature range between them: Madrid and Zaragoza have a continental climate, with a mean temperature very close to that of Barcelona and Bilbao, but with a much wider range. This indicates the existence of latent variables relating to the location, which could play a relevant role in the shape of the relationship between temperature and mortality. On the other hand, when cities were divided into “low”, “medium” and “high-variability” cities on the basis of their coefficient of variation, Madrid and Zaragoza, both classified as “high-variability” cities, showed practically the same pattern marked by a lower risk of mortality.

### 3.2. Temperature Value Associated with Minimum Mortality (MMT)

**Figure 3.**
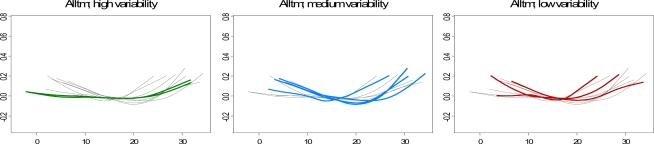

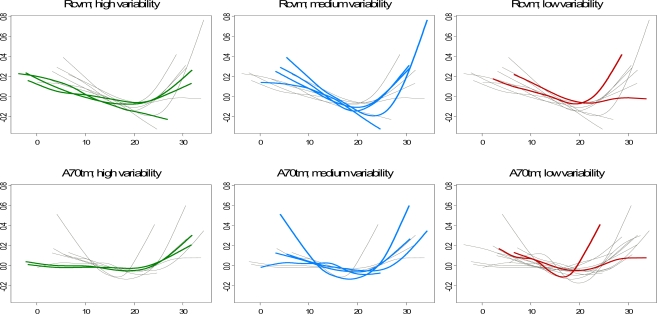
Curves of significant associations between temperature and mortality for: total mortality (Alltm) in the first row, cardio-respiratory mortality (Rcvm) in the second row, and mortality among people 70 years old or over (A70tm) in the third. Cities are grouped according to their Coefficient of Variation into “high variability” (Vitoria, Madrid, Zaragoza), “medium variability” (Oviedo, Vigo, Barcelona, Castellón, Seville) and “low variability” cities (Gijon, Bilbao, Valencia, Huelva, Cartagena). The rest of the curves are drawn in light gray.

**Figure 4.**
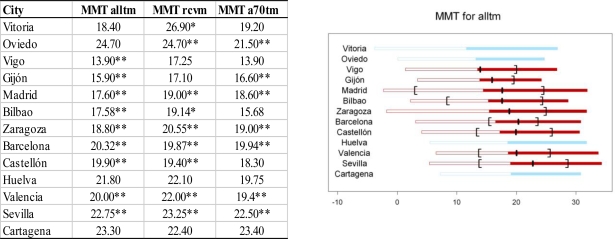

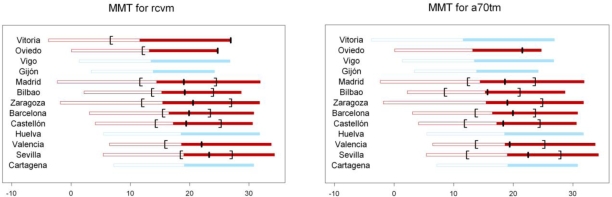
This table includes the temperature associated with minimum mortality (MMT) in each city for total mortality (alltm), mortality due to cardio-respiratory causes (rcvm) and mortality among people 70 years old or over (a70tm). The graphs show: MMT (black segment), the temperature range where the predicted mortality is not statistically different from the minimum mortality (black brackets) and the mean temperature of each city (change from empty to solid box). Red is used for significant associations and blue for non-significant associations. (*) indicates 10% statistically significant associations; (**) indicates 5% statistically significant associations.

The temperature associated with minimum mortality (MMT) for total mortality varied from city to city (14 ºC in Vigo to 23 ºC in Seville) and tended to increase with the mean temperature (Figure 3). The MMT was slightly higher for cardio-respiratory deaths, and the greatest difference with total mortality was around 2 ºC. The graphs in Figure 4 also show that the MMT obtained with these adjusted models remained above the mean temperature. In the three outcomes, the percentage of days with a temperature below the MMT exceeded 50%, and in the case of total mortality this percentage ranged between 60% and 84%. The MMT varied between 13.90 ºC (Vigo) and 22.75 ºC (Seville). We observed a shift of MMT towards higher values as the mean temperature of the cities rises.

In other countries and cities, variable results have been obtained, depending on the climate. For instance, MMTs of around 14 ºC in the south of Finland, 16.5 ºC in Holland, 18ºC in England, 21 ºC in Boston, 27 ºC in Florida and 28 ºC in Taiwan have been reported [3,6,19–22].Different studies have shown that the variation in mortality related to temperature is higher in warm southern countries than in northern ones [23,24]. Even within a country important differences have been found: in England and Wales variations of up to 41% in mortality due to ischemic illness were registered among cities, depending on their different ambient temperature, rainfall and socio-economical differences [25]. In our study, a higher impact was seen in hotter cities

In order to explain these phenomena, several hypotheses have been put forward. On the one hand, there could be a physical process which allows people to adapt to the most habitual temperatures in an area [26]. On the other hand, inhabitants of cities may sometimes adopt, in a secular way, preventive measures to mitigate the impact of temperature variations. These measures are related to the type of housing, the clothes used, or the activities performed in different seasons and at different times during the day [27].

In all cities, the thermal optimum or MMT was found to coincide with a warm temperature for the city, which is broadly consistent with the results found in earlier studies conducted in cities around the world [28–30]. Finally, the comparison with results obtained using a simple model (including only temperature as a predictor) showed that the comfort point moves to the left after controlling for the confounders taken into account. This fact corroborates the importance of considering all the variables which could play a role in the relationship.

### 3.3. Impact of Cold and Heat on Mortality

In general, the effect of heat exceeded the effect of cold. The only exceptions were Valencia for cardio-respiratory mortality and Oviedo for mortality among the population aged under 70 years (Table 2). The effect of cold was greater in hotter cities, showing a correlation coefficient of around 40% with the mean temperature of the cities. The heat effect was also positively correlated with the temperature of cities for specific causes of mortality and mortality in the older population.

Looking at the relationship between the gradient of association and the variability of climate, in general, slopes were negatively correlated with the coefficient of variation, suggesting that the risks are lower in cities with higher variance, in line with the pattern observed in Figure 3.

Finally, there was no significant effect of temperature in the analysis of mortality in people younger than 70 years. In fact, only in two cities, Madrid and Valencia, did the relationship remain significant. In the analysis of mortality due to other causes (non cardio-respiratory ones), there was a significant effect of temperature in six cities. In all of them the impact of cold was lower compared to the impact of cold for cardio-respiratory causes (results not shown).

### 3.4. Differences in the Impact on Specific Causes or Age Groups

A greater effect of temperature, in particular the impact of cold, was found on mortality due to cardio-respiratory causes than on total mortality. The MMTs themselves were higher for specific causes than for total mortality. These results are consistent with those of most published studies. Different hypotheses have been suggested to explain high mortality due to cardiovascular problems in cold months. Among the most plausible ones we find the association between exposure to cold temperatures and a physical reaction resulting in a decrease in blood irrigation to the skin in an attempt to prevent heat loss. This implies an increase in blood volume in the central organs, with a subsequent cardiac overload and an increased blood concentration with higher blood viscosity [31,32]. To some extent, the increase in mortality due to respiratory causes could be due to the impact of certain infectious diseases such as influenza or pneumonia, which present a higher incidence during the cold months [24], and to the increase in fibrinogen concentration related to respiratory infections [32]. As for the increase in cardiovascular mortality due to high temperatures, this has been demonstrated in epidemiological studies [27,33] and related to arterial thrombosis [32].

In the case of mortality in people 70 years old or over, results were very similar to those for total mortality, with a slight increase in the heat effect. This similarity was to be expected given the degree of ageing of the Spanish population: in our series, mortality among elderly people accounts for approximately 70% of total mortality.

Our results show great variability as far as the temperature associated with minimum mortality is concerned. The MMT increases as the mean temperature of each city rises. Classifying the cities into three groups according to their mean temperature—“cold cities”, “mild cities” and “warm cities”—allows for an appreciation of patterns between the curves. A relevant feature is the MMT displacement towards the right (heat) of each curve.

### 3.5. Comparison with Other Studies

Whilst many studies have investigated the relationship between changes in temperature and mortality [5,22,27,34], few have undertaken an analysis similar to the one presented here [6,7,35–38]. Among them only two have been performed in Europe. The first one was conducted in The Netherlands, several years ago [35]. More recently, results from a European multicenter study, including Barcelona and Valencia (the PHEWE Project) [38] have been published [39,40]. These results also showed a U shape in most of the cities. As in our study, greater heat effects were observed in the Mediterranean cities than in the Northern European ones, both for respiratory causes and the elderly [39]. The cold effect was also greater in mild cities and for respiratory causes [40].

The impact of temperature variations on mortality is not negligible. In fact, the effect of a 1 ºC displacement from the MMT is equal to or greater than that associated with a 10 μg/m^3^ increase in the levels of PM_10_. An increase in temperature above the MMT has, in general, a greater effect than a decrease of the same magnitude. For instance, an increase of 5 ºC above the comfort point in Barcelona (20.3 ºC) would be associated with a response of 12.6%, meaning five more deaths a day, while a decrease of the same magnitude would be associated with a response of 7%, or three more deaths a day.

### 3.6. Implications for Public Health

With the evidence currently available, it is difficult to predict how global warming will affect the balance of heat and cold health effects. Some authors consider that there may be a positive effect, consisting in a reduction in the number of deaths in winter that outweighs the extra deaths attributable to the generalised increase in temperatures [19,41]. Other authors provide either positive or negative global estimations, depending on the assumptions they make [42]. Finally, the 4th IPPC Report considered than increase in the number of deaths related to an increase in temperature should outweigh the reduction in deaths expected due to exposure to the cold. However, it is difficult to generalise to all countries. What does seem to be foreseeable is that the increase in number and intensity of unusual episodes (both of heat and of cold), which will presumably accompany global warming, will have a major impact on health. Furthermore, the steeper slope found for changes in heat compared with those in cold make it plausible to expect total increases in mortality as mean temperatures rise. The range of values of MMT found in our study, together with the displacement towards lower values after controlling for confounding, indicates that an increase in temperature, even without reaching extreme situations, may cause an effect on health, as already described in Barcelona [34] and in Valencia [5].

From the point of view of prevention, a series of measures aimed at avoiding or diminishing the effects of temperature variations have been proposed. Such measures comprise short term surveillance actions, such as the implementation of alert systems [43–45] or health education, aimed at health professionals [1], risk groups such as the elderly, children and people with chronic pathologies so that they are informed and able to take adequate personal action in case of extreme temperatures [43]. Ambient corrective actions have also been proposed, such as the adaptation of houses (including heating and air conditioning systems) [46,47]. However, air conditioning should not be the main protective action as it is energy consuming itself, thus contributing even more to the .increase in local and global emissions. Long term climate friendly actions should be preferred as urban planning to mitigate the urban heat island (UHI) phenomenon [48] (temperature increase in cities due to heat emission and obstruction of air circulation caused by buildings) as it could have an important impact on human health. Recent research in Shanghai (China) found heat related mortality was higher in the city centre than in extra-urban locations [49] indicating that UHI may enhance the intensity of heat waves and extreme temperatures, thereby affecting human health due to increased exposure to extreme thermal conditions. Urban planners and local officials should be aware of the increased thermal loads experienced in urban regions and take appropriate action to help reduce the impact of heat on the population.

## 4. Conclusions

To conclude, variations in temperature are strongly associated with mortality. Our study provides results which may help when proposing preventive measures. In any case, further investigation is needed to identify confounders or effect modifiers of the illnesses related to temperature. In this sense, we consider characterisation of the lagged effect, the stratified by seasons study (cold, hot), and the use of combined meteorological variables as a comfort indicator with respect to climatic conditions, *i.e.*, an indicator of the thermal feeling perceived by an individual, to be of great interest for future studies. Lastly, studies which monitor variations in meteorological variables and their relationship with health indicators in places with different climatic, socio-sanitary and ambient features should be undertaken with adequate methods in order to answer specific questions concerning this relationship and to help take preventive actions to minimise the impact of changes in the climatic situation in the short, medium and long term.

## Figures and Tables

**Table 1.** Characteristics of cities.

**City**	**Population**	**n**^a^	**Total mortality**^b^	**Mortality among 70+**^b^	**Cardio-respiratory mortality**^b^	**Temperature**	
						**mean**	**CV**
Vitoria	214,148	1,826	3.53(1.9)	2.38(1.6)	1.58(1.3)	11.59	0.53
Oviedo	198,050	1,461	4.53(2.2)	3.25(1.9)	2.01(1.5)	13.19	0.34
Vigo	274,574	1,461	5.31(2.4)	3.49(2.0)	2.51(1.6)	13.49	0.35
Gijon	261,724	1,096	6.34(2.7)	1.77(1.4)	1.30(1.2)	13.83	0.30
Madrid	2,940,896	1,461	60.82(11.1)	40.47(8.7)	27.83(7.7)	14.43	0.53
Bilbao	667,034	1,461	13.62(4.0)	8.89(3.3)	5.97(2.6)	15.28	0.31
Zaragoza	572,212	1,826	12.49(3.9)	8.94(3.2)	5.80(2.7)	15.45	0.48
Barcelona	1,643,545	1,826	43.62(8.6)	31.04(7.2)	21.23(6.1)	16.49	0.35
Castellón	134,213	1,826	2.9(1.8)	2.15(1.5)	1.58(1.3)	17.23	0.33
Huelva	142,547	1,097	2.59(1.7)	1.77(1.4)	1.30(1.2)	18.53	0.30
Valencia	749,796	1,096	16.09(4.6)	11.1(3.7)	7.73(3.1)	18.61	0.29
Seville	683,028	1,380	13.53(4.3)	8.92(3.4)	6.75(3.0)	19.00	0.33
Cartagena	168,023	1,827	3.47(2.0)	2.47(1.6)	1.77(1.4)	19.07	0.27

a number of days in the series;

b Mean (Standard Deviation).

**Table 2.** Percentage increase in mortality (100 × [exp(b) − 1]) associated with 1 ºC decrease (cold) and 1 ºC increase (heat) in temperature from the MMT for each outcome: total mortality (Alltm), mortality due to cardio-respiratory causes (Rcvm), and total mortality in people 70 years old or over (A70tm). R mean: Pearson correlation coefficient between slopes and mean temperature of cities. R CV: Pearson correlation coefficient between slopes and the Coefficient of Variation of cities.

**city**	**% ∇Alltm**		**% ∇Rcvm**		**% ∇A70tm**	

	**cold**	**heat**	**cold**	**heat**	**cold**	**heat**
**Vitoria**	1.12	1.52	1.86*		1.44	0.99
**Oviedo**	0.96		2.15*		0.68*	0.46*
**Vigo**	1.19*	1.93*	1.55	3.66	1.58	1.37
**Gijón**	0.57*	2.88*	1.31	1.33	2.43*	6.50*
**Madrid**	0.27*	0.93*	1.31*	2.40*	0.38*	2.43*
**Bilbao**	0.85*	1.59*	1.48*	3.74*	1.32	1.86
**Zaragoza**	0.14*	1.39*	0.76*	1.74*	0.06*	1.79*
**Barcelona**	1.41*	2.52*	2.23*	3.42*	1.21*	2.82*
**Castellón**	1.74*	2.60*	3.28*	3.59*	3.95	4.46
**Huelva**	1.11	2.10	2.95	4.17	3.07	4.83
**Valencia**	1.29*	1.44*	1.90*	0.87*	1.44*	1.45*
**Sevilla**	0.88*	2.00*	3.16*	7.72*	0.85*	2.87*
**Cartagena**	1.45	2.16	3.40	4.70	1.32	2.23

***R mean***	*0.43*	*0.05*	*0.46*	*0.40*	*0.37*	*0.48*

***R CV***	−*0.28*	−*0.40*	−*0.52*	−*0.28*	−*0.48*	−*0.01*

(*) indicates a statistically significant association.
